# Identification and characterization of dietary antigens in oral tolerance

**DOI:** 10.1126/sciimmunol.aeb4684

**Published:** 2026-03-06

**Authors:** Jamie E. Blum, Ryan Kong, E.A. Schulman, Francis M. Chen, Rabi Upadhyay, Gabriela Romero-Meza, Dan R. Littman, Michael A. Fischbach, Kazuki Nagashima, Elizabeth S. Sattely

**Affiliations:** 1 Department of Chemical Engineering, Stanford University, Stanford, CA 94305, USA; 2 Howard Hughes Medical Institute, Stanford University, Stanford, CA 94305, USA; 3 NOMIS Center for Immunobiology and Microbial Pathogenesis, Salk Institute for Biological Studies, La Jolla, CA 92037, USA; 4 Department of Cell Biology, New York University School of Medicine, New York, NY 10016, USA; 5 Perlmutter Cancer Center, New York University Langone Health, New York, NY 10016, USA; 6 Howard Hughes Medical Institute, New York University School of Medicine, New York, NY 10016, USA; 7 Department of Bioengineering, Stanford University, Stanford, CA 94305, USA; 8 Department of Microbiology and Immunology, Stanford University School of Medicine, Stanford University, Stanford, CA 94305, USA; 9 ChEM-H Institute, Stanford University, Stanford, CA 94305, USA; 10 Chan Zuckerberg Biohub, San Francisco, CA 94158, USA; 11 Department of Molecular and Cellular Biology, Harvard University, Boston, MA 02138, USA

## Abstract

Food antigens elicit immune tolerance through the action of intestinal regulatory T (T_reg_) cells. Unlike food allergens, the proteins that mediate tolerance are mostly undescribed. Here, we found that epitopes derived from seed storage proteins are targets of murine intestinal T_reg_ cells, with the most frequent response targeting the C terminus of the maize protein alpha-zein. A major histocompatibility complex (MHC) tetramer loaded with this antigen revealed that zein-specific T cells are predominantly intestinal T_reg_ cells, develop concurrently with weaning, and constitute up to 2% of the peripheral T_reg_ cell pool. Zein-responsive T_reg_ cells repressed naïve T cell proliferation ex vivo, and prior dietary exposure resulted in a constrained response upon diverse inflammatory challenges in vivo, supporting a specific role for gut-resident T_reg_ cells in suppressing systemic immune responses. Our work reveals the development, immune-suppressive characteristics, and function of naturally occurring T_reg_ cells that recognize dietary seed storage proteins, a previously undescribed class of antigens in oral tolerance.

## INTRODUCTION

Humans consume nearly 100 g of protein a day from varied sources. Despite being nonself, these foods typically result in oral tolerance, a phenomenon defined by a state of immune unresponsiveness after subsequent exposure to a given antigen (1, 2). Oral tolerance is an intrinsic function of the immune system resulting from continuous surveillance of intestinal contents. Orchestration of oral tolerance and immune suppression is thought to be mediated by regulatory T (T_reg_) cells, a subset of CD4 T cells that recognize distinct dietary epitopes through a unique T cell receptor (TCR) (3, 4). Prior work has pointed to multiple mechanisms for T_reg_ cell–mediated immune suppression, including anti-inflammatory cytokine secretion [e.g., interleukin-10 (IL-10), transforming growth factor–β], competition for growth factors (e.g., IL-2 binding by CD25), and cell-cell inhibition [e.g., lymphocyte activation gene 3 (Lag3), latency associated peptide (LAP), and cytotoxic T lymphocyte associated protein 4 (CTLA4)]. These mediators can suppress conventional T cell activation directly or via reduced antigen presentation (5).

The induction of durable and specific immune tolerance is critical for allergy prevention, and its restoration is the primary goal of allergy immunotherapy. Despite the interest in programming antigen-specific T_reg_ cell responses, few dietary proteins have been identified that mediate tolerance, and none in an untargeted manner that reveals naturally selected epitopes (6). Repertoire-level analyses have shown that antigen-free diets change the abundance and composition of intestinal T cells (3, 7). In one report, nearly half of the peripherally induced T_reg_ cells emerged when mice were exposed to dietary protein and, thus, presumably recognized a food epitope, although specific antigens were not identified (3). Most studies of oral tolerance use model antigens in adoptive transfer paradigms; these antigens differ in dose, route, and timing of exposure, possibly eliciting transient phenotypes; and focusing on an adoptively transferred cell occludes the epitope selection step, which is integral to initiating an immune response (8–12). Recently, in studies to identify immune epitopes from gut-resident bacteria, our team serendipitously identified TCRs responsive to a component of mouse chow (13). Here, we describe the identification and characterization of multiple dietary antigen-TCR pairs and phenotype these food-responsive T cells in vivo to reveal an in-depth profile of the molecular events that result in an oral tolerance response.

## RESULTS

### Identification of dietary antigens from corn, soy, and wheat

To identify the food epitopes recognized by T cells, we adopted a strategy recently developed for the discovery of T cell epitopes from the gut microbiome. In this prior work, mice were colonized with a complex defined bacterial community, intestinal T cells were isolated, and single-cell sequencing was used to identify TCRs (13). We then constructed T cell hybridomas bearing these receptors and performed an in vitro stimulation assay, incubating them individually with each bacterium in the community. We observed four T cell hybridomas that were unresponsive to any of the bacterial strains but were strongly induced by germ-free stool or chow (13), suggesting the possibility that the corresponding TCRs respond to a food-derived epitope. Notably, each of these TCRs was found predominantly (or exclusively) on T_reg_ cells, suggesting that they mediate a tolerogenic response. These data are consistent with our, and previously reported, repertoire-level analyses of mice showing that consumption of protein-containing chow increases the population of gut-resident T_reg_ cells compared with amino acid–defined (AAD) diets (fig. S1) (3).

**Fig. 1.**
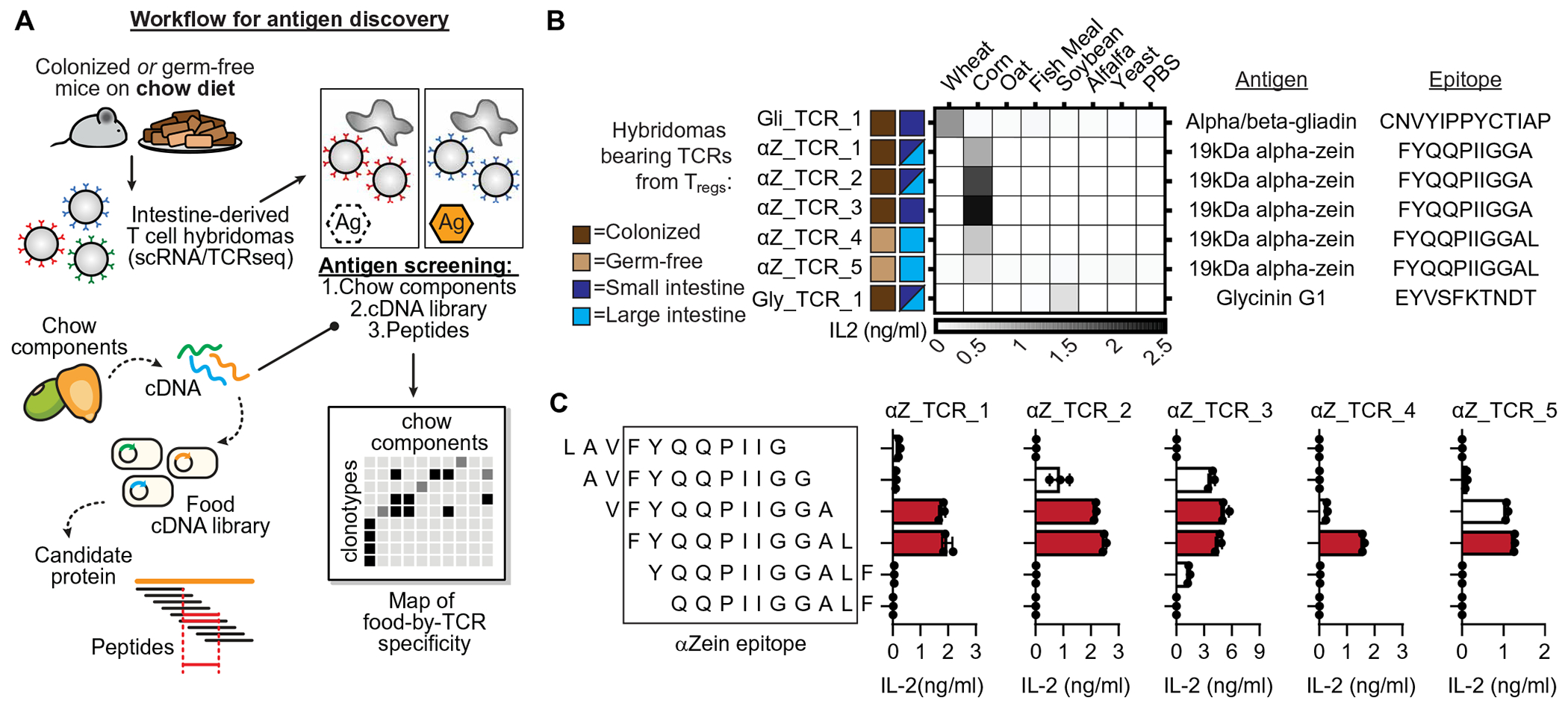
Epitope-TCR pairs identified from wheat, corn, and soy. (**A**) Workflow to screen TCR hybridomas to find food-responsive TCRs from single-cell sequencing data, map the cognate antigens using a cDNA library screen, and map exact epitopes using synthetic peptides. (**B**) TCRs were selected as described in table S1. A mixed lymphocyte assay was used to determine TCR responsiveness to dietary components, antigens, and epitopes. (**C**) Tiling scans with 10– to 11–amino acid peptides spanning αZein were used to identify the minimum antigen epitope that was recognized by each TCR as indicated by IL-2 secretion. Red bars do not differ significantly from each other (*P* > 0.05) and represent the maximum activation. *n* = 3 replicates per condition. *P* values were calculated using a one-way ANOVA with Tukey’s multiple-comparisons test to make all possible pairwise comparisons. Error bars indicate mean ± SD. Every dot represents a cell culture replicate.

In this work, we set out to determine which specific components in chow are recognized by T_reg_ cells as a critical step toward characterizing the molecular events that lead to durable oral tolerance to food encountered through the gut. We first screened TCRs found on sequenced T_reg_ cells selected using a variety of strategies (table S1) for chow responsiveness. In total, 128 hybridoma cell lines each bearing a unique TCR were generated and screened. To identify the antigens recognized by these TCRs, we began by incubating the T cell hybridomas with homogenized preparations of each of the seven protein-containing components of mouse chow (wheat, corn, oat, fish meal, soybean, alfalfa, and yeast) using dendritic cells (DCs) for antigen presentation (Fig. 1, A and B). Five of the TCRs were activated by corn, whereas the other two were specific for soy and wheat. Food-responsive TCRs were cloned from T_reg_ cells obtained from both germ-free and colonized animals, resulting in a total set of seven food-responsive TCRs.

**Fig. 2.**
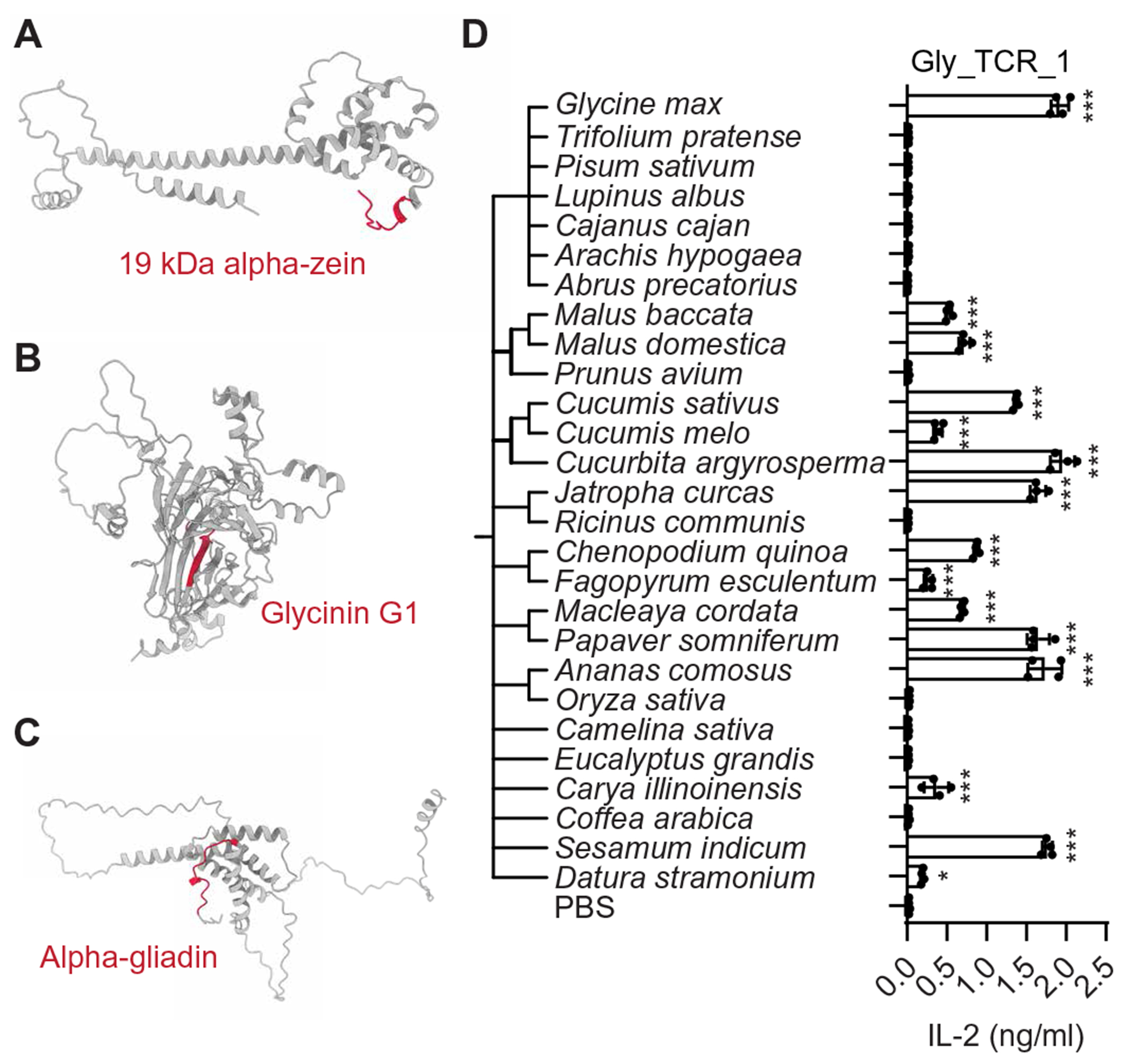
Identified epitopes from αZein, glycinin G1, and gliadin and cross-reactivity of Gly_TCR_1. (**A** to **C**) Alpha-fold predicted structures of αZein, glycinin G1, and gliadin with identified epitopes highlighted in red. (**D**) Gly_TCR_1 was stimulated in the hybridoma mixed lymphocyte assay with soybean or lysates from seeds containing a putative soybean homolog. *n* = 4 per condition. *P* values were calculated using a one-way ANOVA, and Dunnett’s multiple-comparisons test was used to compare each lysate against the PBS control. Error bars indicate mean ± SD. Every dot represents a cell culture replicate. *P < 0.05, **P < 0.01, and ***P < 0.001 compared with the PBS control.

We next used an untargeted approach to identify the cognate antigen of each TCR, starting with the five corn responders. We expressed a corn cDNA library in *Escherichia coli* (Fig. 1A), with the anticipation that this library would approximate the seed proteome [estimated 10,000 proteins (14)]. Approximately 17,280 total clones were selected and initially tested in 576 bins of 30. *E. coli* cells were heat-killed, and the resulting lysates were tested for their ability to activate food-responsive hybridomas. Bins that stimulated a response were retested as individual clones, and those yielding signal were sequenced. All five of the corn-specific TCRs were restimulated by an isoform of the 19-kDa alpha-zein (αZein), a member of a family of closely related maize proteins that differ in sequence and size (15) (TCRs henceforth called αZ_TCRs). We next tested a set of tiled synthetic peptides to identify the epitope recognized from within the protein (Fig. 1A). We found that the C-terminal sequence FY-QQPIIGGAL (αZein_223–233_) was the epitope for all five αZ_TCRs (Figs. 1, B and C, and 2A). In addition to DCs, αZ_TCRs were effectively stimulated by CX3CR1^+^ macrophages, which are known to facilitate antigen sampling in the intestine (fig. S2A) (16). To our knowledge, this epitope has not previously been associated with a T cell response in mice or humans. The αZein_223–233_-responsive TCRs were no more similar in sequence to each other than to other food-responsive TCRs (fig. S2B). The sole exception, αZ_TCR_1 and αZ_TCR_5, differed by only one amino acid. However, they derived from different mice, suggesting independent convergence on a nearly identical TCR sequence. Together, these data suggest that αZein_223–233_ is an immunodominant epitope.

Seed storage proteins were also the targets of the soy and wheat-responsive TCRs. We identified the epitope EYVSFKTNDT from the soy protein glycinin G1 (glycinin_430–439_) using a screening approach similar to that described for corn. The soy epitope resides partially in an internal β sheet of this protein (Figs. 1B and 2B and fig. S2C). This epitope is orthogonal to known glycinin G1 epitopes, which were previously described as antibody-binding regions from patients allergic to soy (17). Further, we found that the wheat-responsive TCR recognizes the recently reported epitope CNVYIPPYCTIAP (gliadin_273–285_) (Fig. 1B and fig. S2D) (6). In prior work in B6 mice, this epitope was found to be a target of the T cell response to gliadin immunization in complete Freund’s adjuvant (CFA), suggesting some degree of peptide-intrinsic dominance across different immune contexts. Like αZein, gliadin belongs to the water-insoluble prolamin group of seed storage proteins, and the epitope is situated within a C-terminal region that is predicted to be unstructured (Fig. 2C).

### Conservation of TCR epitopes across the plant kingdom

Immune cross-reactivity has been well documented between allergens (18–22), but it is unknown whether tolerogenic epitopes also display cross-reactivity. Notably, the gliadin_273–285_ epitope is directly conserved across numerous wheat/grass species, and lysates from these species activated Gli_TCR_1 (fig. S2E and data file S1). A computational search for homologous peptides revealed orthologs of the epitope recognized by the soy-specific TCR in the genomes of many other plants. To test for cross-reactivity, we incubated the Gly_TCR_1 T cell hybridoma with seed lysates from 26 plants, along with DCs. We found that Gly_TCR_1 is robustly activated by seed lysates from numerous plants (Fig. 2D); notable elicitors included other foods such as *Carya illinoinensis* (pecan), *Chenopodium quinoa* (quinoa), and *Sesamum indicum* (sesame seed). Glycinin is an 11S globulin protein, and homologous 11S globulins could explain the cross-reactive signal in other species (data file S2, e.g., 11S globulin seed storage protein 1–like from quinoa with the sequence EWVSFKTND). Orthologs of this epitope are also conserved in foods we did not assess (e.g. walnut, pistachio, and guava) and in trees (e.g., cork oak and white poplar). Curiously, plants that are more closely related to soybean, e.g., *Arachis hypogaea* (peanut) and *Pisum sativum* (pea), were less cross-reactive than more distantly related species.

Although Gly_TCR_1 recognized closely related variants of the soy epitope glycinin_430–439_, the αZ_TCRs were more specific. Synthetic peptides that represent other isoforms of the 19-kDa αZein with similar sequences elicited no response in the same assay (fig. S3A). Seed lysates from *Panicum miliaceum* and *Setaria italica*, which contained predicted αZein_223–233_ homologs, mildly activated only αZ_TCR_3 (fig. S3B and data file S3). It is possible that αZein_223–233_ homologs from other plants are expressed at a low level, different developmental stage, or only under particular environmental/stress conditions. Because processing and presentation of possible epitopes by antigen-presenting cells (APCs) were not assessed, weak or lack of signal in this assay does not exclude potential protein cross-reactivity. To directly test activation in response to a homolog, αZ_TCRs were stimulated with equal quantities of a synthetic peptide from a *P. miliaceum* homolog or the cognate zein epitope. Although these results highlighted the potential for cross-reactivity, they support a preference for αZein_223–233_ (fig. S3C). Thus, together, these findings indicate that some epitopes are directly conserved (e.g., gliadin), some have close cross-reactive homologs (e.g., glycinin), and some have close homologs but exhibit little cross-reactivity (e.g., zein).

### αZein-specific T cells are predominantly small intestine T_reg_ cells

The T cell epitopes we identified are recognized in the physiological setting of a natural food matrix encountered by ingestion, rather than, e.g., a purified protein in drinking water or a food-derived extract administered by injection. Given that the TCRs that recognize these epitopes were predominantly identified on T_reg_ cells, we reasoned that αZein_223–233_-, glycinin_430–439_-, and gliadin_273–285_-responsive T cells may mediate oral tolerance to food.

**Fig. 3.**
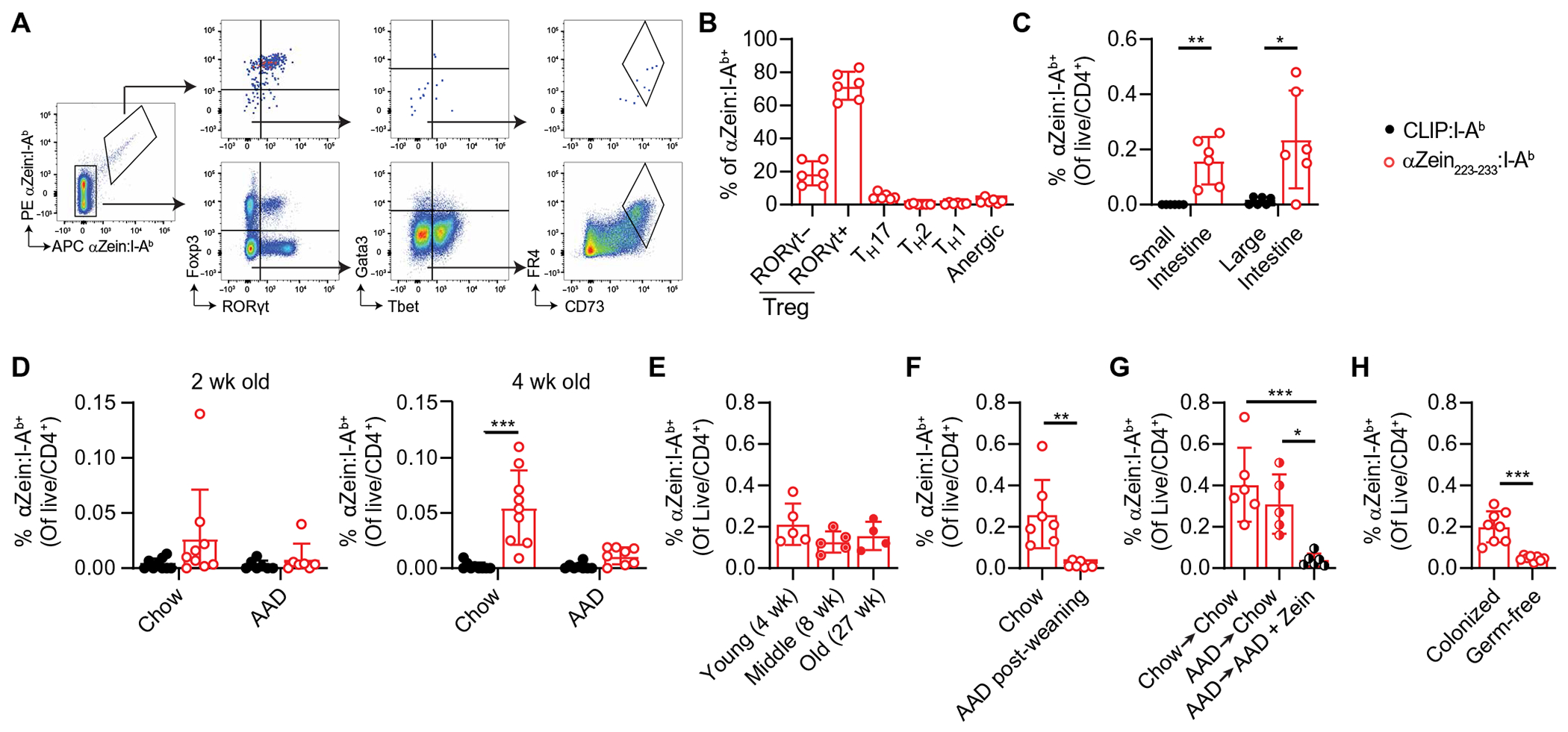
In vivo characterization and factors that influence the abundance of αZein_223–233_-responsive T cells. (**A** and **B**) αZein_223–233_-responsive T cells were profiled for T cell subtypes. RORγt^−^ T_reg_ cells: Foxp3^+^RORγt^−^; RORγt^+^ T_reg_ cells: Foxp3^+^RORγt^+^; T_H_17 cells: Foxp3^−^RORγt^+^; T_H_2 cells: Foxp3^−^RORγt^−^Gata3^+^Tbet^−^; T_H_1 cells: Foxp3^−^RORγt^−^Tbet^+^Gata3^−^; anergic cells: Foxp3^−^RORγt^−^Tbet^−^Gata3^−^CD73^+^FR4^+^. *n* = 6 mice per group. (**C**) Tetramer-positive cells in small and large intestine. *n* = 6 mice per group. (**D**) Tetramer-positive cells in mice born onto chow or AAD diets profiled at 2 or 4 weeks (wk) of age. *n* = 7 to 9 mice per group. (**E**) Tetramer-positive cells in mice at different ages. *n* = 4 or 5 mice per group. (**F**) Tetramer-positive cells in mice swapped from chow onto an AAD diet at weaning or maintained on a chow diet, profiled at 12 weeks of age (9 weeks on AAD diet). *n* = 7 mice per group. (**G**) Tetramer-positive cells in mice on chow diet or mice born on AAD diet then swapped onto chow or AAD + 10% zein for 2 weeks, from 6 to 8 weeks of age. *n* = 5 or 6 mice per group. (**H**) Tetramer-positive cells in colonized or germ-free adult mice. *n* = 8 mice per group. All analyses are pregated on live CD4^+^ cells. (D) includes data from male and female mice; all other panels use female mice only. *P* values were calculated using a paired *t* test (C), two-factor ANOVA (D) with a Šidák’s multiple-comparisons test to follow up on significant interaction terms, one-factor ANOVA [(E) and (G)] with a Tukey’s multiple-comparisons test, or unpaired *t* test [(F) and (H)]. Unless otherwise indicated, all T_reg_ cells were analyzed from small intestine laminae propriae of chow-fed mice. Every dot represents an individual mouse. Error bars indicate mean ± SD. *P < 0.05, **P < 0.01, and ***P < 0.001.

To characterize the distribution and functional properties of αZein_223–233_-responsive T cells, we obtained a major histocompatibility complex II (MHCII) tetramer loaded with αZein_223–233_, the corn-derived epitope recognized by five of the seven TCRs we mapped. We first analyzed the context in which αZein-specific T_reg_ cells emerge. In young adult (6 to 12 weeks old) mice fed a standard chow diet, small intestine αZein_223–233_ tetramer–positive T cells were predominantly Foxp3^+^ T_reg_ cells, and a majority expressed the transcription factor RORγt across mice from two different vendors (Fig. 3, A and B, and fig. S4A). αZein-specific T cells were also detected in the large intestine (Fig. 3C). Compared with the small intestine, fewer numbers of αZein-specific T cells were detected in the large intestine, precluding definitive analysis of cell type distribution, although some T_reg_ cells were observed (fig. S4B). On average, greater than 2% of small intestine peripheral T_reg_ (pT_reg_) cells (Foxp3^+^Helios^−^ or Foxp3^+^RORγt^+^ cells) were tetramer positive, a strikingly large response against a single food epitope (fig. S4C). This result suggests the possibility that a subset of food epitopes dominate immune recognition in the context of tolerance. αZein_223–233_-specific T cells were also detected in the mesenteric lymph nodes, although to a much smaller degree than in the intestine (fig. S4D).

Although prior work has established the kinetics of pT_reg_ cell induction postbirth, the development of food-responsive T cells is less well understood, limiting our knowledge of the window during which tolerance develops. Food tolerance most likely arises either soon after birth, supported by the detection of food peptides in breast milk (23), or concomitant with weaning and the introduction of solid foods. To assess these possibilities, dams were randomized during pregnancy to a chow or an AAD (antigen-free) diet. Their pups were fed the same diet until they were euthanized at 2 or 4 weeks of age. Tetramer-positive T cells appeared at 4 weeks of age only in the chow-fed mice (Fig. 3D and fig. S5A), mirroring an increase in total pT_reg_ cells across weaning (fig. S5, B and C). In other studies, pT_reg_ cells were detected in the intestine 12 days after ovalbumin (OVA) exposure, suggesting that initial priming may have occurred around 2 weeks of age (10).

Immunoglobulin G (IgG) antibodies against specific food components developed in parallel to food-responsive T cells, highlighting that food protein exposure activates both the cellular and humoral branches of the adaptive immune system (fig. S6, A and B). These food-targeting antibodies constituted a substantial portion of the total serum IgG, persisted throughout life contingent on continued chow exposure, and were also detectable in a panel of serum samples from healthy human donors (fig. S6, C to E). Although the role of IgG in oral tolerance and the relationship between T_reg_ cells and antibodies are generally not well understood, there is some evidence that IgG contributes to systemic food tolerance (24, 25). Together, these data show that the first introduction to food is a critical window for development of food-responsive pT_reg_ cells, which appears to co-occur with an increase in IgG antibodies that recognize food components; both arms of immune recognition of food appear to reach a steady state that lasts through adulthood.

After weaning, the abundance and subtype distribution of αZein-specific T cells were stable across a range of ages (Fig. 3E and fig. S7A). As expected, this stability depended on continued chow exposure; mice swapped onto an AAD diet after weaning experienced a sharp reduction in αZein-specific T_reg_ cell abundance (Fig. 3F). When mice born onto an AAD diet were introduced to chow after weaning, the αZein-specific T cell response developed normally, although feeding a zein protein fraction without other chow components induced a much weaker response (Fig. 3G and fig. S7B). This finding suggests a dependency on context of zein exposure (e.g., potential exposure to other food molecules and/or physical food matrix) for development of αZein-specific T_reg_ cells, an aspect of food T_reg_ cell development, which is not shared with purified OVA (10). Further highlighting the importance of intestinal context, in germ-free mice, the abundance of αZein-specific T cells was markedly reduced (Fig. 3H), and the phenotype was shifted toward fewer RORγt^+^ and more RORγt^−^ T_reg_ cells (fig. S7C), revealing a potential interplay between immune development, gut microbiota, and food.

The identification of RORγt^+^Foxp3^+^ T_reg_ cells specific for αZein suggests a possible parallel between food-responsive and bacterially induced T cells, given that commensals such as *Helicobacter hepaticus* (*Hh*) induce the same “double-positive” T_reg_ cells (26). Induction of these *Hh*-responsive T_reg_ cells depends on RORγt^+^ APCs (27). Similarly, we found that MHCII^ΔRORγt^ mice, which lack MHCII expression on RORγt^+^ APCs, have fewer RORγt T_reg_ cells and a corresponding increase in T helper 17 (T_H_17) cells responsive to both αZein and gliadin (fig. S8). These results suggest similarities in requisite APCs between peripheral antigens of diverse origin (bacteria and food).

### Immunosuppressive factors are altered in food-responsive T_reg_ cells

To search for molecular features that are characteristic of naturally induced food-responsive T cells, we measured the transcriptional profile of αZein-specific T_reg_ cells. In one experiment, we used single-cell RNA sequencing to profile αZein-specific T_reg_ cells, *Helicobacter*-responsive T_reg_ cells, and bulk controls (all T_reg_ cells regardless of antigen-specificity) (figs. S9 to S11). In a parallel experiment, bulk RNA sequencing was used to compare αZein-specific T_reg_ cells with adoptively transferred OVA-specific T_reg_ cells (after exposure to OVA in drinking water) and bulk T_reg_ cells (all CD4^+^CD25^+^ cells of unknown antigen specificity) from chow-fed, AAD-fed, or germ-free mice (fig. S12).

**Fig. 4.**
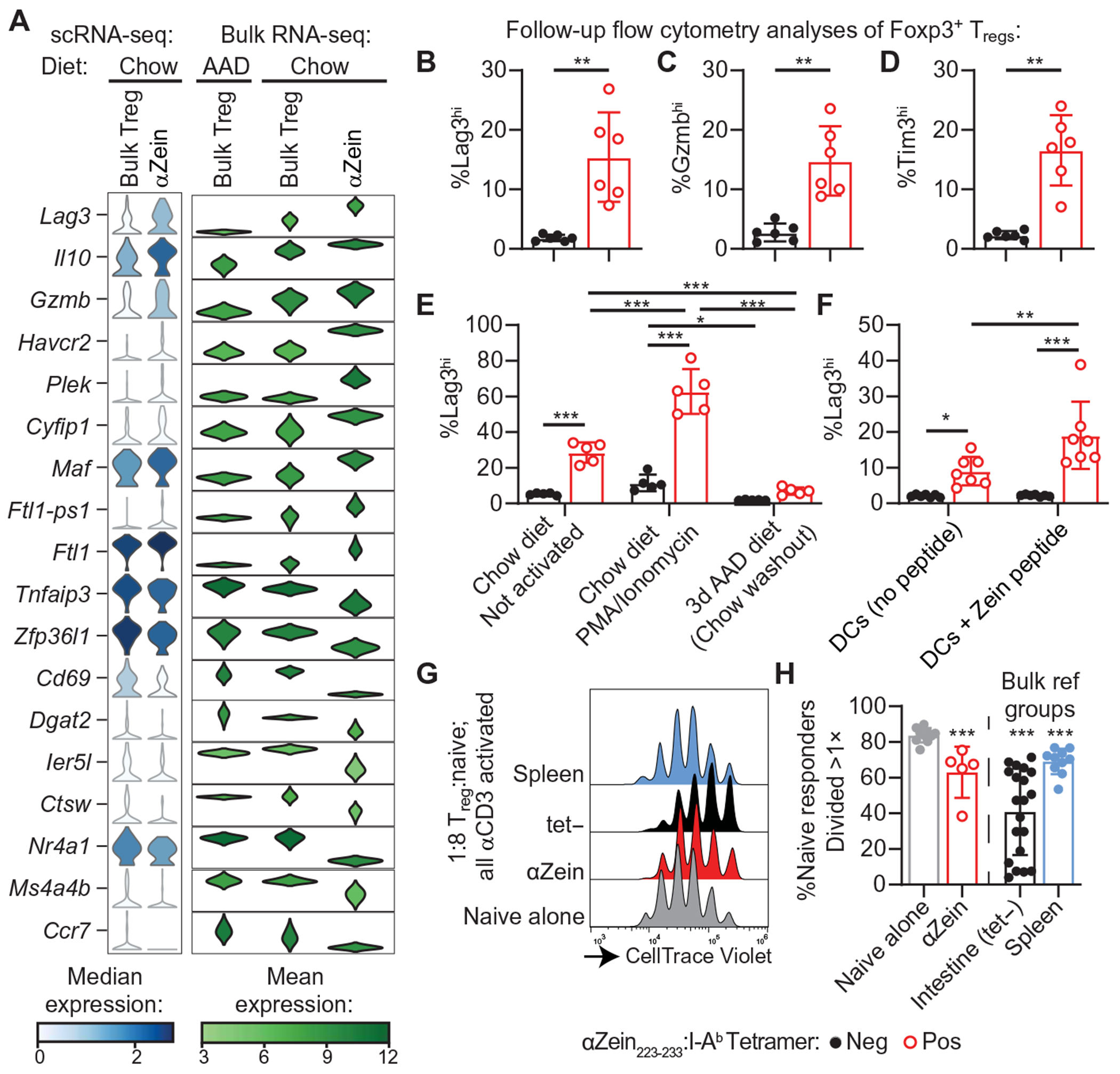
Food-responsive T_reg_ cells display a distinct transcriptional signature of immune-suppressive markers. (**A**) Violin plots showing a selection of differential genes of interest shared in both the single-cell and bulk sequencing datasets. Single-cell data compare bulk T_reg_ cells from chow-fed control mice with antigen-specific αZein_223–233_ T_reg_ cells, and the bulk sequencing data compare bulk T_reg_ cells from chow-fed control mice, AAD-fed mice, or antigen-specific αZein_223–233_ T_reg_ cells. (**B** to **D**) Lag3, Gzmb, and Tim3 levels between αZein_223–233_ binding and tetramer nonbinding T_reg_ cells. *n* = 6 mice per group. (**E**) Lag3 levels between αZein tetramer-positive and tetramer-negative populations, under control conditions, activation by a 4-hour PMA/ionomycin stimulation, or 3 days on AAD diet compared with control chow-fed mice. *n* = 5 mice per group. (**F**) Lamina propria single-cell suspension cocultured for 4 hours with DCs (no added peptide) or DCs pulsed with zein peptide (FYQQPIIGGAL). *n* = 7 mice per group. (**G** and **H**) Percentages of naïve T cells divided after incubation with APCs, a T_reg_ cell population (either αZein-specific T_reg_ cells, tetramer-negative T_reg_ cells, or splenic T_reg_ cells), and αCD3 antibodies. *n* = 10 naïve controls, 5 Zein T_reg_ cells, 20 intestinal tet–T_reg_ cells, and 10 spleen T_reg_ cell samples. Statistical analysis was used to compare each group against the naive alone control. (B) to (F) were gated on live CD4^+^Foxp3^+^ and αZein:I-Ab-PE^+^αZein:I-Ab-APC^+^ (tetramer-positive) or αZein:I-Ab-PE^−^αZein:I-Ab-APC^−^ (tetramer-negative). (G) and (H) were gated on live CD4^+^CD45.1^+^CTV^−^. *P* values are calculated using paired *t* test [(B) to (D)], two-factor ANOVA [(E) and (F)] with a Tukey’s post hoc test or uncorrected Fisher’s LSD test, or unpaired *t* test (H). Every dot represents an individual mouse. Error bars indicate mean ± SD. *P < 0.05, **P < 0.01, and ***P < 0.001.

Several genes associated with immune suppression (e.g., *Gzmb* and *Lag3*) emerged from the sequencing data as associated with food responsiveness (Fig. 4A and data file S4). Follow-up flow cytometry assays revealed distinct profiles of immune-suppressive markers (e.g., Lag3, Gzmb, Tim3, and CTLA4) in αZein-specific T_reg_ cells, other food-antigen specific T_reg_ cells, and across bulk T_reg_ cell populations from mice with different environmental antigen exposures (AAD diet and germ-free) (Fig. 4, B to D, and figs. S13 to S15). Recently, some of the same immune-suppressive genes were also identified as up-regulated in intestinal epithelium–resident T_reg_ cells from chow compared with AAD diet–fed mice, suggesting a conserved effect in multiple intestine layers (7). Our tetramer data corroborate this finding and specifically implicate food-responsive T cells as a source of these differences. We considered activation status as a possible explanation for the observed differences, either reflecting underlying T_reg_ cell biology or as an artifact of tetramer staining. Prolonged tetramer staining can lead to T cell activation, thus altering phenotype (28); however, experiments with OVA suggested that our tetramer staining protocol did not alter the immune-suppressive molecule profile (fig. S16). Further, our single-cell RNA sequencing data did not show enrichment of canonical T_reg_ cell activation markers in αZein-specific T_reg_ cells compared to bulk controls, and across all T_reg_ cells, there was no apparent relationship between multiple immune-suppressive and activation-associated transcripts (fig. S17, A and B) (29–31). Although activation status at the time of cell isolation is unlikely to explain differences in the transcript profile, intentional ex vivo activation did exacerbate differences in immune-suppressive programs between αZein-specific and bulk T_reg_ cells. Lag3 expression was induced in intestinal αZein-specific T_reg_ cells by chemical [phorbol 12-myristate 13-acetate (PMA)/ionomycin] or peptide stimulation and diminished after a short-term Zein washout (3 days on AAD diet) (Fig. 4, E and F). These data reveal that the transcriptional profile of αZein-specific T_reg_ cells is dynamic and influenced by a combination of factors, including recency of antigen exposure and activation by external elicitors.

To further assess cell state, we compared our single-cell sequencing data against published T_reg_ gene expression profiles. Compared with bulk intestinal T_reg_ cells, αZein-specific T_reg_ cells aligned more closely to tissue-adapted “effector T_reg_ cells” or “IL-10^stable^ T_reg_ cells,” suggesting that habitual zein exposure induces a stable tissue-resident T_reg_ cell population (32, 33) (fig. S17, C to E). We tried to leverage the observed single-cell gene expression profile to identify additional food-responsive TCRs. This effort revealed an additional gliadin_273–285_ responder, highlighting the immunodominance of this epitope and some capacity for gene expression profile to reveal antigen specificity. However, this approach was not a systematic improvement over other mapping strategies (table S1 and fig. S18).

To more directly assess the suppressive capacity of food responsive T_reg_ cells, we sought to measure αZein-specific T_reg_ cell–mediated suppression of naïve T cell expansion after an ex vivo αCD3 stimulation. Isolated αZein-specific T_reg_ cells effectively suppressed division of naïve T cells (Fig. 4, G to H). We observed that the suppressive capacity of the bulk T_reg_ cell pool was highly variable and also changed depending on diet and anatomical site, likely reflecting the distinct arsenal of immune-suppressive factors expressed by each population (figs. S19 and S20). For example, although chow T_reg_ cells from the lamina propria were Lag3^hi^ and Gzmb^hi^, consistent with our RNA sequencing data, these cells simultaneously expressed low levels of other immune-suppressive makers such as Klrg1 and PD-1. Together, these findings indicate that αZein-specific T_reg_ cells adopt a phenotype reflective of a mature tissue-resident cell state and enriched in select immune-suppressive factors. These data provide direct evidence that T_reg_ cells responding to an immunodominant food epitope engage in canonical suppression programs (34–36) and that a single T_reg_ cell can concurrently use multiple suppressive strategies. Furthermore, these suppressive programs appear to be up-regulated by recognition of the cognate food epitope.

### Oral consumption elicits systemic zein tolerance

We next evaluated contexts in which αZein-specific T_reg_ cells are immunomodulatory in vivo. First, we measured antibody levels after an inflammatory intraperitoneal sensitization, a model which robustly reveals tolerance in mice previously exposed to oral OVA (fig. S21, A and B). Unexpectedly, no anti-zein antibodies were observed after intraperitoneal zein sensitization in mice consuming either a defined corn-containing (AAD + corn/soy/wheat/oat) or corn-free (AAD + soy/wheat/oat) diet (fig. S21, C and D). When mice on the same diets were exposed to intraperitoneal OVA, OVA antibodies were robustly detected (fig. S21E). To explore this further, we injected chow- or AAD-fed mice with whole corn extract (fig. S22A). In this model, both corn- and zein-targeted antibodies were higher in AAD mice compared with chow-fed mice, consistent with a protective effect of chow (fig. S22, B and C). Moreover, MHCII ^ΔRORγt^ mice, which had fewer RORγt^+^ T_reg_ cells responsive to αZein, also showed higher levels of corn- and zein-targeted antibodies compared with wild-type controls despite all mice consuming chow diet (fig. S22, D to F). Collectively, these data reveal that in conditions where αZein-specific T_reg_ cell development is impaired, either through diet or genetic knockout, oral tolerance to systemic challenge is also less effective. To further investigate oral tolerance to a complex lysate, a similar model was used to probe oral tolerance toward sesame (fig. S22G). Given that we observed cross-reactivity between the TCR we identified as reactive to glycinin G1 with sesame lysate, we included a soy-containing diet as a control group. Sesame consumption induced oral tolerance to sesame and soy consumption provided protection compared with an AAD diet, revealing potential protective effects of antigen cross-reactivity (fig. S22H). However, we cannot rule out a role for diet complexity or a diet containing any protein in explaining protective effects of soy.

As an orthogonal approach to measuring zein allergy, we used a cholera toxin–driven oral sensitization model. This model robustly revealed OVA allergy but provided no evidence of zein or gliadin allergy in mice born on an AAD diet (fig. S23, A to D). To better understand the relationship between antigen and allergy development in this model, we further tested casein and lactoglobulin, two milk proteins with different biochemical properties. Although some of the lactoglobulin-sensitized mice experienced anaphylactic allergy, none of the casein-sensitized mice displayed symptoms (fig. S23, E to H). These data suggest that inflammatory responses to proteins (including zein) depend on the context and perhaps also the identity of the protein. However, these data also emphasize a protective effect of oral corn exposure to subsequent immune responses to corn, consistent with an immune-suppressive nature of αZein-specific T_reg_ cells.

**Fig. 5.**
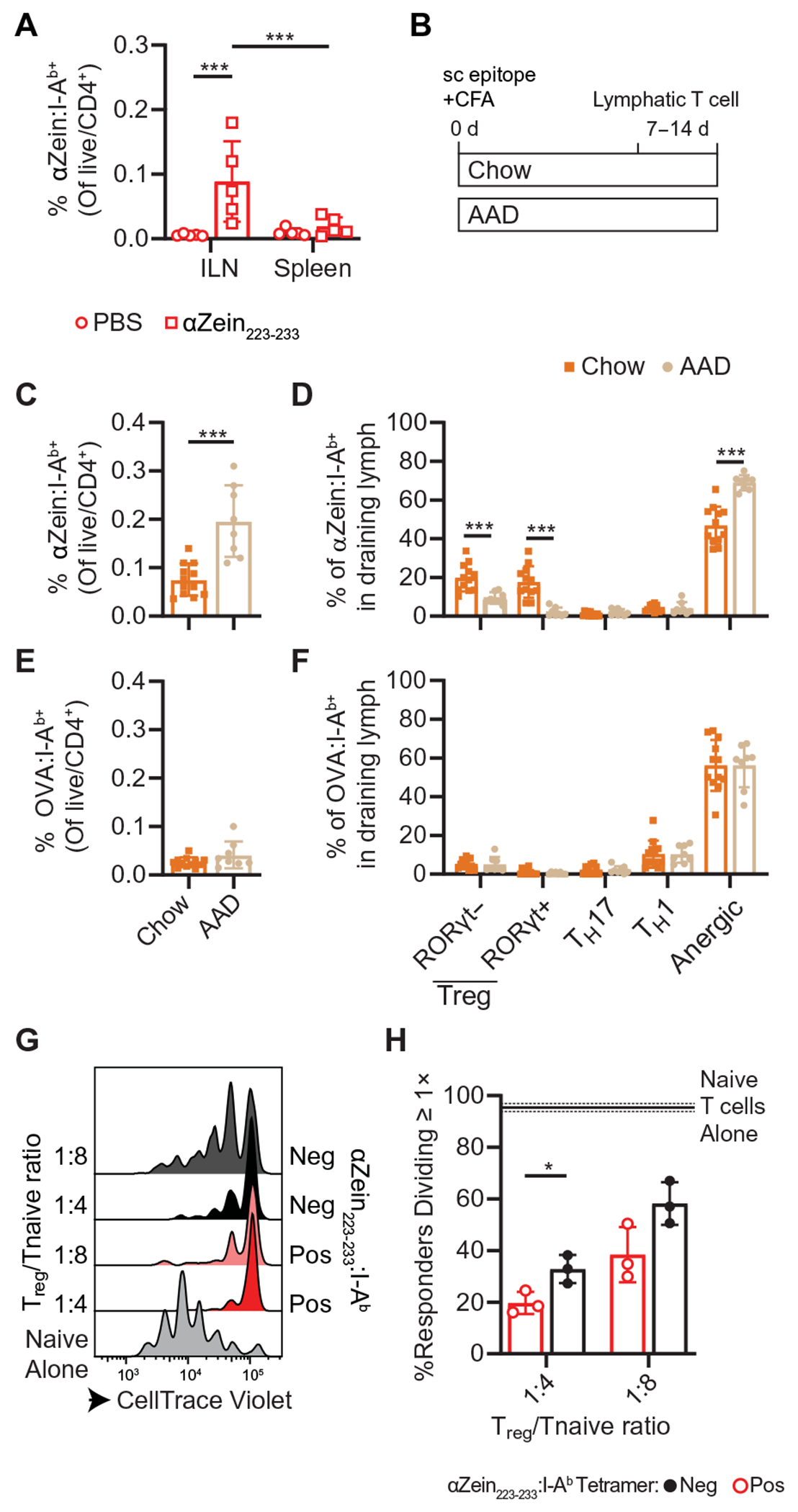
αZein-specific T cell profile and suppressive capacity after CFA simulation. (**A**) Tetramer-positive cells in the inguinal lymph node (ILN) and spleen after an intraperitoneal injection with CFA or zein epitope emulsified in CFA. *n* = 5 mice per group. (**B**) Mice were born onto chow or AAD diets. In adulthood (6 to 10 weeks), mice were given a subcutaneous injection of zein epitope emulsified in CFA on day 0. T cells were isolated from the draining lymph nodes between 7 and 14 days postinjection. (**C** to **F**) Abundances and distributions of cell types responsive to αZein or OVA in draining lymph nodes after epitope + CFA injection. *n* = 8 to 12 mice per group. Only significant pairwise comparisons within a cell type are shown. (**G** and **H**) Percentages of naïve T cells divided after incubation with APCs, a T_reg_ cell population (αZein-specific T_reg_ cells or tetramer-negative T_reg_ cells isolated from the inguinal lymph node), and αCD3 antibodies. *n* = 3 mice per group. (C) and (E) were gated on live CD4^+^TCRβ^+^, (D) was gated on live CD4^+^TCRβ^+^αZein:I-Ab-PE^+^αZein:I-Ab-APC^+^, and (F) was gated on live CD4^+^TCRβ^+^OVA:I-Ab-PE^+^OVA:I-Ab-APC^+^. *P* values were calculated using a two-factor repeated-measures ANOVA with an uncorrected Fisher’s LSD test (A), an unpaired *t* test [(C), (E), and (H)], two-factor repeated-measures ANOVA with a Šidák’s multiple-comparisons test (D), or two-factor repeated-measures ANOVA (F). Every dot represents an individual mouse. Error bars indicate mean ± SD. *P < 0.05, **P < 0.01, and ***P < 0.001.

To more directly probe the function of T cell–mediated zein tolerance, we used a CFA-driven inflammation model (37). Injection of zein epitope emulsified in CFA induced expansion of αZein-specific T cells in the inguinal lymph node (Fig. 5A). To determine the role of oral tolerance in modulating this response, we compared mice born onto chow diets with existing zein tolerance with mice born onto AAD diets for which this was the first zein exposure (Fig. 5B). Consuming chow diet reduced the frequency of the CFA-induced αZein-specific T cells in the draining lymph and skewed the T cell response to include more T_reg_ cells and fewer anergic cells (Fig. 5, C and D), consistent with a tolerogenic immune-suppressive response. This effect was dependent on dietary exposure to the specific epitope, as evidenced by the observation that the T cell response to a nondietary epitope (OVA) was the same on chow or AAD diet (Fig. 5, E and F). Correspondingly, when isolated lymph node cells were stimulated with zein peptide ex vivo, IL-2 production, a marker of T cell activation, was constrained in samples from the chow-fed mice (fig. S24A). Dietary background did not affect T cell activation for an unrelated antigen (2W1S; fig. S24B). To directly assess the function of the Zein-responsive T_reg_ cells from chow-fed mice, we tested their immune-suppressive capacity against naïve T cells. CFA-expanded αZein-specific T_reg_ cells isolated from the inflammatory lymph node setting displayed robust immune suppression (Fig. 5, G and H). Further, these lymph-derived αZein-specific T_reg_ cells from chow-fed mice displayed a Lag3^hi^ phenotype comparable to that observed in intestinal αZein-specific T_reg_ cells (fig. S24C). Blocking Lag3 alleviated some of the αZein-specific T_reg_ cell–mediated suppression, suggesting a causal role for Lag3 in αZein-specific T_reg_ cell–mediated suppression (fig. S24, D to F).

As a final measure of T_reg_ cell function, we adoptively transferred αZein-specific T_reg_ cells into naïve recipient mice and measured the sufficiency of these T_reg_ cells to mediate immune tolerance. To achieve necessary numbers of αZein-specific T_reg_ cells and total cells for transfer, we used CFA-expanded (with or without zein epitope) total CD4^+^CD25^+^ T_reg_ cells from chow-fed mice as the donor cell population (fig. S25, A and B). The activity of these T_reg_ cells was then tested in the recipient mice using a subcutaneous epitope + CFA challenge model, with T cell abundance and phenotype as the readout. This model was adapted from a similar assay previously used to show tolerance toward self-antigens and enabled discrimination of adoptively transferred and host T cells (38). Transfer of αZein-specific T_reg_ cells suppressed the generation of αZein-specific T_H_1 cells in recipients (fig. S25, C to F). The frequency of newly induced Zein-specific T cells and IL-2 secretion in response to ex vivo restimulation was unaffected by the presence of adoptively transferred αZein-specific T_reg_ cells, which could suggest specific suppression of T_H_1-driven inflammation in this model (fig. S25, G and H). Overall, these data reveal that zein tolerance achieved through chow consumption facilitates T_reg_-mediated suppression of a future inflammatory challenge toward zein.

## DISCUSSION

Oral tolerance is a remarkable process of suppressing immune responses toward dietary proteins. Our ability to characterize the development of oral tolerance and mechanisms of T_reg_ cell–mediated immune suppression has been limited because the antigens that are natural T_reg_ cell ligands were unknown. Here, we identified food epitopes that are recognized by naturally induced T_reg_ cells after oral introduction of antigens. The antigens identified, αZein, glycinin, and gliadin, are all seed storage proteins, suggesting that this class of proteins is a common source of tolerogenic epitopes. We found that αZein-specific T_reg_ cell emergence depends on several factors, including developmental state and intestinal context (microbiome, food matrix, and sampling mechanisms). Further, we demonstrated that food-responsive T_reg_ cells are characterized by a distinct transcriptional profile, including up-regulation of a subset of immune- suppressive molecules. These immune-suppressive agents were previously reported as differential across bulk T_reg_ populations from chow and AAD-fed intestinal epithelial or lamina propria cells but not differential between a gut microbe–specific T_reg_ cell population and bulk controls, confirming a specific association with food-reactive T_reg_ cells (3, 7, 39). Last, we demonstrated the functional immune-suppressive capacity of αZein-specific T_reg_ cells and their role in oral tolerance. Notably, oral exposure to corn under conditions that elicit αZein-specific T_reg_ cells protects against an inflammatory response to corn driven by intraperitoneal immunization. Adoptive transfer experiments with αZein-specific T_reg_ cells revealed the specific role of these cells in promoting oral tolerance. We anticipate that our findings regarding oral tolerance toward zein, as an abundant and stable dietary protein, will directly translate to other seed storage proteins, including those that are prevalent in food allergy.

Our characterization of zein tolerance, combined with other model studies of oral tolerance, begins to highlight potential contributions of the specific antigen to observed immune outcomes. High-dose OVA in drinking water coupled to adoptively transferred OTII cells is a robust and widely used oral tolerance model. In contrast, dietary zein alone only weakly induced T_reg_ cells compared with zein incorporated in chow. Intraperitoneal OVA, not but zein, strongly induced antibodies. These data suggest a possible role for the food matrix in promoting immune recognition of zein through both the oral and intraperitoneal routes. T cell phenotype is also antigen and context dependent. For example, although chow consumption induced a predominant αZein-specific T_reg_ cell population, after 1 week on a gliadin-containing diet, many gliadin-responsive T cells adopted an anergic phenotype (6). In other studies, after short-term OVA exposure, only half of adoptively transferred OVA-specific T cells become Foxp3^+^ T_reg_ cells (3, 10). Determining whether these differences reflect antigen-intrinsic properties or experimental parameters (dose, duration, age at initial exposure, etc.) is essential to understand the range of possible tolerance phenotypes. T_reg_ cell populations induced by short-term feeding could be functionally distinct from long-term gut-resident T_reg_ cells, and the functional consequence of phenotype for durable tolerance is unknown. Thus, identifying and characterizing chow-responsive T cells are essential to understand the nature of long-standing oral tolerance.

It is notable that the food T_reg_ cell epitopes we have found from two different common dietary grains, including the immunodominant tolerance antigen from corn, derive from highly abundant, water-insoluble proteins. Furthermore, corn is widely tolerated in human populations and does not commonly result in food allergy. Our data suggest that this lack of a negative immune response is not due to limited access of corn proteins to intestinal immune cells. In contrast, our measurement of αZein-specific T_reg_ cells indicates that corn proteins are effectively sampled and presented by gut-resident APCs. In contrast with the proteins we identified as sources of T_reg_ cell antigens, proteins that are known to be recognized by T_H_2 cells in food allergy are largely water soluble. Together, these observations further underscore that physical properties coupled with relative abundance of key proteins in food might directly affect whether a given food is likely to be widely tolerogenic versus susceptible to immune sensitization. Further experiments are needed to determine whether solubility or other biochemical factors are key determinants of pathogenic immunogenicity. This unexpected outcome of our work is worth future investigation because it could help explain why some foods are disproportionately associated with increased incidence of food allergies. Identifying tolerance epitopes has direct implications for understanding allergies. For example, glycinin G1 is a known allergen (Gly m 6). However, the epitope we identified does not correspond to a known allergen epitope [from the immune epitope database (IEDB) (17, 40–46)], with the caveat that IEDB consists primarily of human epitopes. In addition, the glycinin-responsive TCR is not activated by known Gly m 6 cross-reactive lysates [e.g., peanut lysate/allergen Ara h 3 (47, 48)]. In the future, tracking T cell responses to this epitope under inflammatory or homeostatic conditions may reveal vulnerabilities that initiate allergy development. Further, understanding how proteins like αZein, that are not common allergens, drive strong T_reg_ cell responses could provide a blueprint for programming antigen-specific T_reg_ cell responses. Cereal grains comprise more than half of the world’s daily caloric intake, and we anticipate that the proteins identified here will also be common targets of T_reg_ cells in humans (49). Our analysis of human serum samples begins to provide evidence for immune recognition of multiple dietary grains, including corn.

Diet is our most intimate interaction with our environment. Correctly recognizing foods as safe creates an anti-inflammatory environment to support nutrient acquisition and prevent allergy. This research advances our understanding of the major dietary antigens recognized by intestinal T_reg_ cells and demonstrates their function in oral tolerance toward prolamin antigens. Tapping mechanisms of oral tolerance as naturally occurring programming with molecular specificity could enable the use of synthetic approaches for redirecting allergic and autoimmune states.

## MATERIALS AND METHODS

### Study design

This study was designed to identify antigens from mouse diet that are recognized by T_reg_ cells following homeostatic chow consumption. First, we generated hybridoma cell lines bearing TCRs identified on T_reg_ cells from intestinal single-cell RNA sequencing datasets. These hybridomas were screened using an in vitro mixed lymphocyte assay. For receptors that were reactive to a food component, we mapped the specific antigen epitope and determined cross-reactivity toward other plant extracts. One epitope from the maize protein αZein emerged as immunodominant and was prioritized for further study. An MHCII tetramer loaded with this epitope was used to characterize the abundance, location, development, and persistence of food-responsive T_reg_ cells. Zein-reactive T_reg_ cells were further characterized using both bulk and single-cell RNA sequencing. Last, we used numerous in vitro and in vivo models to assess the capacity of αZein-specific T_reg_ cells to perform immune suppression and mediate oral tolerance. Sample sizes were determined on the basis of prior experience. Mice were randomized to studies and no mice were excluded. Blinding was not performed.

### Tissue samples from mouse and human

All studies were conducted under administrative panel for laboratory animal care approved protocols at Stanford (protocol 33997) or institutional animal care and usage committee protocols through the New York University School of Medicine (NYU) and adhered to ethical standards for the treatment of research mice. Animals were housed in a conventional facility with 12-hour light–12-hour dark cycles. When indicated, mice were fed AAD AIN-93G diet (Dyets Inc., Item 510017). Experiments at Stanford were performed with C57BL/6 animals from Jax (strains: 002014 or 000664) or Taconic (B6-F). For the development study, timed pregnant C57BL/6 mice were purchased from Jax. Mice were randomly maintained on chow diet or switched onto the AAD diet upon arrival and gave birth within 1 week. For all other studies of mice born on AAD diets, C57BL/6 mice were bred in-house on AAD diet starting when breeders were set up. When indicated, custom diets were made by mixing L-AA–defined AIN93G diet with other human-grade food ingredients at the indicated percentages. Germ-free mice were maintained in aseptic incubators.

Ptprc^a^Pepc^b^/BoyJ mice were purchased from Jax and given 1 week to acclimate to the animal care facility. On the first day of the study, 1 × 10^6^ OTII cells were adoptively transferred from Rag2/OTII mice (strain, 11490; Taconic) by retroorbital injection. Mice then received OVA (10 mg/ml; Sigma-Aldrich) in drinking water for 7 days. At NYU Grossman School of Medicine, all transgenic mice were bred and maintained in the Alexandria Center for Life Sciences–West Tower vivarium in specific pathogen–free conditions. C57BL/6 mice (Jax no. 000664), I-AB^f/f^ (B6.129X1-H2-Ab1tm1Koni/J Jax 013181) mice were purchased from Jackson Laboratories. RORγt-*cre* and Hh-72tg were generated by members of the Littman laboratory and have previously been described (26, 50). Female and male mice were used equally in this experiment. Mice from 6 to 12 weeks of age were used. All mice were housed with a 6 a.m.-6 p.m. light on-off cycle with an ambient temperature of 18° to 24°C and humidity maintained between 30 and 70%. Human serum samples were obtained from the Stanford Blood Center from anonymous donors.

### Intestinal T cell isolation

Small intestine was removed from the animal and adipose tissue and Peyer’s patches were carefully removed. A sagittal cut was made through the lumen, opening the intestine into a flat layer. Intestinal samples were incubated at 37°C in a solution of Dulbecco’s modified Eagle medium [DMEM (VWR)] + 5% fetal bovine serum (FBS; Thermo Fisher Scientific) with 5 mM EDTA (Sigma-Aldrich) and 1 mM dithiothreitol (Thermo Fisher Scientific) for 40 min with shaking. Next, samples were placed in conical tube with warm DMEM and shaken vigorously for ~20 s. Samples were collected on a strainer and the shaking step was repeated once more. Samples were then processed on a gentleMACS (Miltenyi Biotec) using the LPDK-1 program and enzyme solutions from the lamina propria dissociation kit (no. 130-097-410, Miltenyi Biotec). When samples were finished on the MACS, an equal volume of 80% Percoll PLUS density gradient media (Cytiva) was added, diluting the sample to 40% percoll. The sample was moved to a new conical tube, and 80% percoll was added to the bottom using a transfer pipette, resulting in two layers. The samples were centrifuged at 600*g* for 10 min at room temperature, and cells were recovered from the interface of the 40 and 80% percoll layers. Cells were washed in 10 ml of media and then passed through a filter before preparing for flow cytometry analysis.

### Flow cytometry

All tetramers were provided by the NIH Tetramer Core Facility with both PE and APC fluorophores. The following tetramers were used: αZein (FYQQPIIGGAL), CLIP (PVSKMRMATPLLMQA), Gliadin (NVYIPPYCTIAP), and OVA (AAHAEINEA and HAAHAEINEA). The two OVA tetramers were used as a pool. Tetramer staining was performed for 1 hour at 37°C in Roswell Park Memorial Institute ([RPMI], Thermo Fisher, 61870127) media with 10% FBS or for 1 hour at room temperature in MACS buffer with 5% FBS at a 1:100 dilution. To enhance detection of antigen-specific T cells in the CFA-peptide experiment, cells were treated with a protein-kinase inhibitor, dasatinib (MedChemExpress, HY-10181), at a final concentration of 50 nM for 30 min before and throughout tetramer staining at 37°C. Cells were stained with viability dye, and in some experiments cell surface markers, in MACS buffer (Miltenyi Biotec) with 5% FBS, then fixed and permeabilized using the eBioscience Foxp3/Transcription factor staining buffer set (Thermo Fisher Scientific, 00-5523-00). Cells were fixed for either 1 hour at room temperature or overnight at 4°C. Intracellular targets, and in some experiments intracellular and cell surface targets, were stained in permeabilization buffer. All antibodies used are indicated in table S2. At Stanford, cells were analyzed using an LSRII or Symphony (BD Bioscience) analyzer and sorted using either a Sony SH800 or a FACSAria II at the Stanford Shared FACS Facility. Flow cytometry experiments at NYU were performed on an Aurora (Cytek) analyzer, while cells were sorted using a FACsAria (BD Bioscience). All flow cytometry data were analyzed using FloJo software.

### Hybridoma generation

Hybridomas were generated as previously described (13). In brief, pMSCV-mCD4-PIG TCR-OTII backbone was used in combination with TCRα/TCRβ sequences of interest (data file S5), which were separated by a P2A peptide and synthesized commercially (Twist Bioscience) or cloned in lab. αZ_TCR_1, αZ _TCR_2, αZ _TCR_3, and Gly_TCR_1 had been generated and found to be food responsive previously (13). All other hybridomas were generated in this study. Lipofectamine 3000 reagent (L3000001, Thermo Fisher Scientific) was used to generate virus particles containing the TCR vector in Platinum-E cells (RV-101, Cell Biolabs). The virus was then used to transduce NFAT-GFP hybridoma cells. The presence of a functional TCR in all hybridomas was confirmed by measuring IL-2 secretion after stimulation with an anti-CD3 antibody. Some hybridomas were selected on the basis of clonal relationship identified using GLIPH2 (51).

### T_reg_ cell suppression assay

CD45.2^+^CD4^+^green fluorescent protein (GFP)^+^αZein_223–233_:I-Ab^+^ T_reg_ cells and CD45.2^+^CD4^+^GFP^+^αZein_223–233_:I-Ab^−^ T_reg_ cells were sorted from the small intestine lamina propria of Foxp3-GFP reporter mice (B6.Cg-Foxp3tm2Tch/J, Jax strain, 006772). CD45.2^+^ CD4^+^GFP^+^ T_reg_ cells were also sorted from spleens. For some experiments, T_reg_ cells were instead isolated from draining lymph nodes 11d following CFA-peptide sensitization, following the methods described below. Naïve CD45.1^+^ T cells were isolated from spleen by pre-enrichment using a naïve CD4^+^ T cell isolation kit (Miltenyi Biotec), stained with CellTrace Violet dye (CTV; Thermo Fisher Scientific), and subsequently purified by flow cytometry (live/CD4^+^/CD62L^+^/CD44^−^). T_reg_ cells were cultured at a ratio ranging from 1:32 to 1:2 (determined on the basis of number of T_reg_ cells sorted) with naïve T cells in the presence of 1 μg/ml anti-CD3 (BioLegend) and DCs isolated as described above) at a 1:1 DC–to–T cell ratio in a V-bottom plate. Naïve T cells cultured alone with anti-CD3 and DCs were used as a control. In some experiments, cells were treated with anti-Lag3 (30 μg/ml; BioXCell, BE0174) or antiIsotype control (30 μg/ml; BioXCell, BE0088). Cell proliferation was measured after 3 days of coculture by measuring CTV dilution in the CD45.1^+^ T cell population. During the assay, cells were cultured in RPMI 1640 + 10% FBS + 1% penicillin-streptomycin + 1× sodium pyruvate (Gibco) + 1× MEM Nonessential amino acids (Gibco) + 50 μM BME (VWR).

### Intraperitoneal allergy models

For measuring OVA tolerance, C57Bl/6 mice were given water (not tolerized) or water supplemented with OVA (10 mg/ml; Sigma-Aldrich) (tolerized) ad libitum for 1 week. Two days after ceasing supplemented water feeding, both groups of mice were intraperitoneally injected with 100 μl of a solution containing 50 μg of OVA precipitated with 1 mg of Imject Alum (Thermo Fisher Scientific) in phosphate-buffered saline (PBS). Mice were given a booster dose in a similar fashion 2 weeks after the initial injection with 100 μl of a solution containing 50 μg of OVA in PBS. Serum was collected 1 week after the second injection and assayed for anti-OVA IgG1 at a 1:6000 dilution using a commercially available enzyme-linked immunosorbent assay (ELISA) kit (Cayman).

For zein tolerance, in the first experiment, mice had three dietary backgrounds: born and maintained on chow; born on AAD and randomized at 4 weeks of age to start a defined corn-containing diet (AAD diet + 10% each of corn, soy, wheat, and oat); born on AAD and randomized at 4 weeks of age to start a defined corn-free diet (AAD diet + 10% each of soy, wheat, and oat). At 6 weeks, mice began the inflammatory injection series which consisted of an initial dose of 500 μg of protein + 1 mg of Imject Alum (Thermo Fisher Scientific) on day 0, a booster dose of 50 μg of protein on day 14, and serum collection posteuthanasia on day 21.

In the parallel experiment to measure zein tolerance, mice born onto chow or AAD diets were sensitized with intraperitoneal corn. At 6 weeks of age mice began the inflammatory exposure series consisting of a 500-μg dose of corn + 1 mg of Imject Alum (Thermo Fisher Scientific) on day 0, booster with 50-μg corn on day 14, and serum collection posteuthanasia on day 21. The same experimental series was repeated with MHCII ^ΔRORγt^ mice and control mice fed chow diets.

### Oral allergy model

To induce allergy, mice were sensitized to zein (Santa Cruz Biotechnology), gliadin (Sigma-Aldrich), OVA (Sigma-Aldrich), casein (Santa Cruz Biotechnology), or β-lactoglobulin (Sigma-Aldrich) alongside 10 μg of cholera toxin (Millipore) in 200 μg of 5% sodium bicarbonate two times (days 0 and 7). On day 14, mice were challenged with an intraperitoneal injection of 2 mg of cognate allergen. Body temperature was measured every 5 min for 1 hour using the Bio Medic Data Systems transponder system.

### Peptide administration in CFA

In the initial experiment, mice were injected intraperitoneally with 100 μg of zein epitope emulsified in CFA. The spleen and inguinal lymph node were harvested after 7 days. In subsequent experiments, mice were subcutaneously injected in the right flank or tail base with 100 μl of CFA (Invivogen) emulsion containing 10 μg of each epitope of interest. Peptides (2W1S: EAWGALANWAVDSA; OVA: ISQAVHAAHAEINEAGR; Zein: FYQQPIIGGAL) were purchased from Genscript. Draining inguinal and axillary lymph nodes were harvested 14 days postinjection and processed into single-cell suspensions which were then stained with relevant tetramers and antibodies specific for phenotypic markers as described previously. Draining inguinal and axillary lymph nodes were harvested 14 days postinjection and processed into single-cell suspensions, which were then stained with relevant tetramers and antibodies specific for phenotypic markers as described previously. In experiments to probe the suppressive capacity of Zein T_reg_ cells, only the zein epitope was injected. As an orthogonal readout of activation, mice were injected with 10 μg of Zein or 2W1S epitope. On day 8, single-cell suspensions were isolated from the inguinal lymph node and restimulated ex vivo with cognate peptide (10 μg/ml). After 24 hours, media IL-2 levels were measured using an ELISA assay as described above.

### Statistics

Statistical analysis was performed in GraphPad Prism 9. Comparison of two groups was performed with a *t* test and comparison of three or more groups with a one-factor analysis of variance (ANOVA). Data with two variables were analyzed using a two-factor ANOVA. When data were collected from paired samples, for example, tetramer-positive and negative data from the same mouse, a paired *t* test was performed. Tukey’s multiple-comparisons test, Dunnett’s multiple-comparison test, Šidák’s multiple-comparisons test, or the uncorrected Fisher’s LSD test was used for post hoc analysis of ANOVA data. *P* < 0.05 was considered statistically significant.

## Supplementary Material

SupplementaryFiles

Data files S1 to S6

MDaR Reproducibility checklist

## Data Availability

Sequencing data generated in this study are deposited in EMBL-EBI (European Molecular Biology Laboratory’s European Bioinformatics Institute) ArrayExpress under accession number E-MTAB-16143 (bulk RNA sequencing) and E-MTAB-16270 (single-cell RNA sequencing). Tabulated data underlying all of the figures are provided in data file S6. All other data needed to support the conclusions of the paper are present in the paper or the Supplementary Materials. Code is available at Zenodo (52).Tetramer reagents unique to this research were provided by the NIH Tetramer Core Facility (NIH Contract 75N93020D00005 and RRID:SCR_026557) and are available from them upon request. Hybridoma cell lines were generated by the authors and are available from J.E.B. under an MTA (material transfer agreement) from the Salk Institute. RORγt-*cre* and Hh-72tg mice are available from Jax (stocks 032538 and 022791). All other materials are commercially available as described in the Materials and Methods.
